# Are delay ages at marriage increasing? Pre-marital sexual relation among youth people in the place of residence in India

**DOI:** 10.1186/s12905-022-02149-3

**Published:** 2023-01-11

**Authors:** Ujjwal Das, Sasmita Rout

**Affiliations:** grid.444315.3 0000 0000 9013 5080 P.G. Department of Geography, Fakir Mohan University, Balasore, Odisha India

**Keywords:** Adolescent, Marriage, Wealth quintile, Education, Sexual intercourse

## Abstract

**Background:**

Adolescent sexual and reproductive health is a major public health issue throughout the world. At the same time shifting of marriage are undergoing discernible changes in country like India. This paper attempts to examine the effect of delay age at marriage on the risks of pre-marital sexual intercourse for the youth people in the place of residence.

**Methods:**

Data used in the present study is from various annual publications of Sample Registration System (SRS) and four round of National Family Health Survey, which was conducted in 2015–2016. The Kaplan–Meier life table technique and multivariate regression models are used to examine the premarital sex by the place of residence and marriage cohort.

**Results:**

Findings of the study indicate that the reasons underlying delayed marriage differs between blow 21 years age group and 22–30 years age group. Multinomial analysis clearly shows education, wealth quintile and mass media are major controlling factors of delayed age at marriage. Residing in urban adolescent women who belonged to better economic family background and exposed to mass media had a higher probability to experience premarital sexual intercourse than the rural adolescent in delay age group.

**Conclusion:**

The study concludes that the restorative the empowerment of youth especially for women and health care provider should consider a multidimensional approach for higher education among youth people and safe sexual behaviour in pre-marital sexual intercourse.

## Introduction

Marriage is a universal process, but recent trends indicate that there is increase in the age at marriage in the country. Government of India has changed the age at marriage for women at 21 in the year 2021. Further, A comparative analysis between 2001 and 1991 census data clearly explains that for those who have been married during 1992–2001, marriages remain at higher ages as compared to those married for twenty years or more, before 1991 census., Hence, the pattern of delayed marriages in India has remained relevant and needs further enquiry. The prior research observed that marriage patterns are based on much more similarities in union formation, such as “the similarities between partners regarding their social class, level of education, employment, religion, ethnic group and family background” [1]. In addition, marriages get delayed for lack of proper matches and at the same time decision-making power of the youth people play the dominant role of individual marriage. However, the levels of difficulties lies in identifying the possible factors that may explain why the youth people remain unmarried or get married in the delayed age group. Simultaneously, increase in the delayed age of marriage among the youth people is because of the widening sexual behaviour among the young couple in the place of residence. Because the adolescence age group (15–19) is one of the most critical transitions in the life course. “This period is part of rapid biological, cognitive, and psychosocial development, encompassing not only the physical changes of puberty but also leading to the development of sexual maturity” [2]. In addition, “the global adolescent population (10–19) is estimated to have reached 1.3 billion (49%: 15–19 years old) in 2020, of whom over 235 million (46%: 15–19 years old) live in sub-Saharan Africa (SSA), accounting for 23% of the region’s population” [3].

Furthermore, delayed marriage has now become a major problem, which may lead to a “change in the sequence of sexual initiation and marriage” [4]. In addition, delayed marriage has brought the issues such as “dating, premarital sexual relations, unwanted pregnancies”, thereby increasing likelihood of the spread of HIV/AIDS [5]. As a consequence because of extensive gap between puberty and marriage there is “an increase in likelihood that young women will become involved in sexual relationships before marriage” [6]. Prior research on pre-marital sex in the United State of America, Finer (2007), concluded that “premarital sex is not surprising in an era when men and women typically marry in their mid to late twenties and they are sexually active as singles for extensive periods” [7]. On the contrary, due to pre-marital sex females are mostly affected with associated diseases like sexually transmitted diseases HIV/AIDS as compared to their male counterparts. Alo and Akinde (2010) suggested that the sexually transmitted diseases HIV/AIDS are higher among the females than the males as a result of premarital sex [8]. Also, “first-time sexual intercourse increases the possibility of contracting sexually transmitted infections (STIs) because people do not have appropriate knowledge of the precautions to protect from STIs” [9]. The early age of premarital sexual behaviours may be lead to a high risk of adolescent pregnancy, “sexually transmitted diseases (STDs), prevalence of illegal and unsafe abortions, single mother–child/abandoned child and juvenile delinquency” [10, 11].

Several literatures have identified association of “individuals, family structures, peer groups, and community-level factors with pre-marital sexual experiences among young people” [12–14]. Further, it is found that at the individual level, education and pre-marital sex is negatively correlated [15, 16], and at the family level, factors such as “co-residence with a parent, parental disapproval of pre-marital sexual activities, and parental connectedness” used to prevent or delay pre-marital sexual initiation among young people especially strongly associated with young women [17, 18]. Further “good, open, and higher levels of parent–child communication play an important role in lowering the rates of premarital intercourse with partners among young people [19].

The sexual variations between men and women in the place of residence are substantial. For women, the median age at first intercourse is low in rural residences because of early marriage and high in urban residences [11]. For men, age at first intercourse is not linked to age at marriage while it is found that in most African and Asian countries, men started to have sex later than women [20]. In addition, “in some industrialized countries, sexual activity before the age of 15 years has become more common in recent decades” [9, 21]. Furthermore, the trend towards later marriage in many countries have also explains the reasons for an increasing prevalence of premarital intercourse. It is also found that the trends in the average time spend between first sexual intercourse and settling with a marital or cohabiting partner are different among men and women. “The time between first sexual intercourse and living with a partner is longer for men than for women (typically 3–6 years compared with 0–2 years respectively)” [22]. This might also be explained by the age-sex pattern of sexual activity. The existing literatures have suggested that in the younger age groups (15–19) women are highly engaged in sexual activity while the case is reverse in older age groups (≥ 20 years). Most importantly, a large proportion of men have more than one sexual partner, whereas the women are predominantly monogamous. It is also noticed that the age of marriage has been increasing because of increasing influence of education and economic empowerment of both gender in place of residence. Hence, “a substantial portion of boys and girls in contemporary India have to deal with a long period of sexual desire” [23]. Premarital sexual relationships among the youth people are more common in urban residences as compared to the rural residence, because of urban lifestyle behaviour, decision making power influence the delayed age at marriage as well as premarital sex in higher age group (≥ 20 years) [10, 24, 25].

The socio-cultural phenomena play an important role in increasing premarital sex. Better nutrition and better health care facilities lead to attain puberty at early age. While “Indian girls started their first puberty at the ages between 8 and 13 and reach menarche (first menstruation) early, boys on contrary enter puberty between the ages of 9 and 14” [26]. With early puberty girls are vulnerability to early marriage in the developing countries [27] and therefore entering into sexual life [22]. Thus the possible factors such as declining age at puberty and the increasing age at marriage may have created an opportunity for premarital sexual relationships [28]. Both aspect timing and romantic partner relationship have immediate individual effects in premarital relationships among the youth people. Women who have intercourse before marriage or at very young age have to face serious health problems such as unplanned pregnancy which may lead to unsafe abortion [29]. Therefore, the study aims to understand the tendency of premarital sex among the youth people with the effect of delayed age at marriage in the residences.

## Data and methods

In this study the data is used from the fourth round of the National Family Health Survey (NFHS)-4, conducted under Ministry of Health and Family Welfare (MoHFW), Government of India for the year 2015–2016. The survey provides information on demographic and health indicators at the national, regional, state, and district levels from a nationally representative sample. NFHS-4 (2015–2016) collected information from a nationally representative sample of 601,509 households, and 699,686 women of age 15–49. The present study deal with the premarital sexual behaviour among the youth people due to delay age at marriage in India, and the analysis is based on information of married and unmarried people from rural and urban residence in ages 15–30 years youth people. In rural areas total information was collected from 176,282 among them 171,956 are married and 4326 are unmarried, while in urban residence information was covered total 59,376 among them 57,418 are married and 1958 are unmarried of age 15–30 years respectively [30].

Furthermore, various annual publications of the Sample Registration System (SRS) are used for identifying the level, trend, and regional variation of female age at marriage in the country. Further, it is to be noted here that the Sample Registration System was introduced as a pilot scheme in some selected Indian states in 1964–65 to generate reliable estimates of nuptiality, fertility, and mortality at the national and state levels, which strengthens the reasons for using such data sources. All the data analysis in this study is done by using STATA version12.0 software.

### Outcome variable

The premarital sex is the main outcome variables in the study. The measurement of premarital sex is a very crucial and sensitive issue. For which National Family Health Survey did not ask the question directly to the respondents about premarital sex. For the absence of variable on premarital sexual intercourse, we have analysed premarital sex among married and unmarried men and women at age 30 years. And those are currently unmarried and had experienced sex, as a considered pre-marital sex which was also confirmed earlier study done by Melesse et al. [31]. To note here, premarital sex is combing measures of the age and first vaginal sexual intercourse. As the present study intends to define premarital sex for the first time, therefore, respondents were asked a few questions, such as, “Have you ever had sexual intercourse?“ If so, “how old were you when you had your first sexual intercourse? For additional information on age at marriage: “Have you ever been married?“ and “How old were you when you first got married?“ from the analysis it is understood that the individuals who had sex a month or more earlier than their month of the first marriage, or who had had sex but had not married by the time of the interview, were considered to have experienced pre-marital sex. Those who had sex for the first time in the same month as (or after) their first marriage and those who had neither had sex nor married contributed their months of non-experience of the premarital sex and were “censored” at the time of marriage (for those who had married) or at the time of interview (for those who had not married), since they ceased to be at risk of the event at that point. After that life table procedure was used for censored information, which is considered as the best suitable method [7].

### Independent variables

Several studies have highlighted the demographic and community variables, which are explaining premarital sex in various countries [32–37]. The main independent variable of this study is age at marriage in 30 years, it was divided into three categories’, such as married early (less than 21 years), married late (27–30 years) and unmarried till 30 years. The others variables are included age, education, residence, caste, religion, region, wealth index, and regular exposure to mass media (television, radio, and newspaper) as independent variables.

### Statistical methods

Bivariate and multivariate analyses were used for the analysis of premarital sex due to delayed age at marriage. The life table technique was used to measure the median age at premarital intercourse. For multinomial analyses, the relative risk ratio (RRR) was estimated for the measurement of premarital sex in various ages. Multinominal discrete-time models with a logit transformation [38] are used to understand the effects of key independent variables on the probability of premarital intercourse. The relative risks of premarital intercourse can be analysed with a general formulation as follows:

$${\text{Logit}} \left[\text{P}\left(\text{Y}=1\right)\right]={\upalpha }+{{\upbeta }}_{I}{\text{x}}_{I}+{{\upbeta }}_{2}{x}_{2}+\cdots +{{\upbeta }}_{k}{\text{x}}_{k}$$

And the alternative formula, directly specifying π (x), is

$$\pi \left(x\right)=\frac{\exp(\alpha +{{\upbeta }}_{I}{\text{x}}_{I}+\cdots +{{\upbeta }}_{k}{\text{x}}_{k})}{1+\exp(\alpha +{{\upbeta }}_{I}{\text{x}}_{I}+\cdots +{{\upbeta }}_{k}{\text{x}}_{k})}$$

where the P represents the risk of premarital intercourse at the age of youth people, the parameter ${{\upbeta }}_{I}$refers to the effect of${\text{x}}_{I}$, on the log odds that Y = 1, controlling other${\text{x}}_{j}$, for instance, $\text{e}\text{x}\text{p}$ (${{\upbeta }}_{i}$) is the multiplicative effect on the odds of one unit increase in${\text{x}}_{i}$, at fixed levels of other${\text{x}}_{j}$ .

We have *n* independent observations with p-independent variables i.e. age at marriage, and the qualitative response variables were k categorical, constructed the logits in the multinomial case. If the Relative Risks Ratio (RRR) is greater than 1, then indicates that with increasing values on the independent variables there is an increased likelihood /risk of a case falling into the comparison category and decreased risk of falling into the baseline category. Contrary to that if RRR is less than 1, then it indicates that with increasing values on the IV there is decreased risk of a case falling into the comparison group and an increased risk of the case falling into the baseline category. If the RRR equals 1, then there is no relationship between the IV and the risk of falling into the comparison group with the baseline group. And also note that if ${\upbeta }$ =0, then RRR = 1. If ${\upbeta }$>0, then RRR>1, if ${\upbeta }$<0, then RRR <1 respectively. All analysis has been done by using the statistical software package STATA® (Version 14.0).

## Results

Table 1 represents the mean age of female marriage by place of residence in India from 2011 to 2018. Within 7 years’ time period female age at marriage increased by 1.1 years, wherein in 2011 it was 21.2 years and in 2018 it was 22.3 years respectively. Similarly, the same trend was also found in rural residences but the urban residence female age at marriage increased by 0.7 (22.7 to 23.4)) months from 2011 to 2018. It indicates that the delayed age at marriage is relatively higher in urban residences as compared to rural residences. Because in urban areas most of the families belonged to the richest quintile, and they are supported in higher education, which leads to delayed marriage in the residences.

**Table 1:** Mean age at effective marriage of female by place of residence

Year	Total	Rural	Urban
2011	21.2	20.7	22.7
2012	21.2	20.8	22.4
2013	21.3	21.0	22.5
2014	22.3	21.8	23.2
2015	22.1	21.6	23.0
2016	22.2	21.7	23.1
2017	22.1	21.7	23.1
2018	22.3	21.8	23.4

*Sources*: Various Annual reports in SRS

**Fig. 1:**
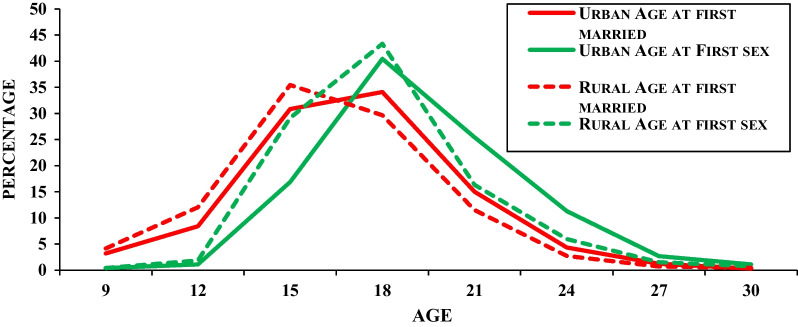
Percentage distribution of adolescents who had first sexual intercourse, marriage by specific age in place of residence

Figure 1 shows the proportion of individuals in the 2015–2016 survey who had premarital sex and married by specific age in the place of residence. Table 2 contains the proportion of youth who had premarital sex by specific age for all respondents by gender and place of residence. By the exact age of 18 years, 40% of premarital sex in urban residences and 44% have in rural residences. In that age group, 34% had married in urban residences and 29% had married in rural residences respectively. In the age group of 21 years, 11% had married in rural residences and 16% in urban residences. The premarital sex of the corresponding age was found at 16% and 24% in rural and urban residences respectively. Thus it indicates that delay in age at marriage and premarital sex was higher in urban residents as compared to rural residents. “Although the overall marriage curve is included for comparison to the sex curves, the percentage who had premarital sex by a certain age cannot be calculated by taking the difference between the sex curve and the marriage curve at that age because most of those who both had sex and been married by that age had sex first”, which is also confirmed by Finner, in his earlier study [7].

**Table 2:** Percentage of various groups who had premarital sex by specific ages in place of residence

Place of residence	Percent who had premarital sex by exact age:								
	Group	9	12	15	18	21	24	27	30
Urban	All	0.41	1.11	16.88	40.45	25.44	11.31	2.7	1.11
	Male	0.53	1.13	19.22	41.15	24.34	10.1	2.26	0.68
	Female	NA	1.06	8.71	37.99	29.29	15.57	4.22	2.64
Rural	All	0.43	1.87	29.14	43.33	16.63	5.97	1.53	0.75
	Male	0.44	1.9	29.7	44.0	16.31	5.54	1.33	0.51
	Female	0.38	1.71	26.05	39.54	18.44	8.37	2.66	2.09

*NA* Not available

**Fig. 2:**
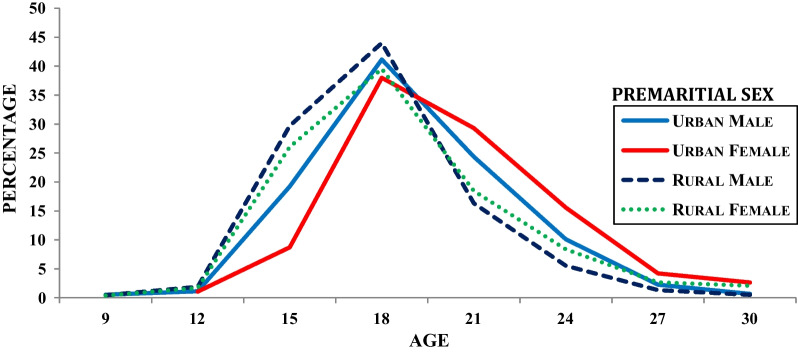
Percentage of individuals who had premarital sex by specific ages and sex in place of residence, 2015–2016

Figure 2 presents the gender-wise differential of premarital sex in the place of residence. It is found that in the age group below 18 years male and female premarital sex of urban residents were 19% and 9% respectively. The corresponding figures found in rural residents were 29 and 26% respectively. Moreover, in the age group of 21 years premarital sex in rural residences was 16 and 18% for both males and females. On the contrary, the premarital sex in the urban residents in the same age group for both males and females was 24 and 29% respectively. Further, after the age group of 21 years, prevalence of premarital sex declined but the gender-based difference was still constant for both residences. It indicates that males were slightly more likely to have premarital sex at the early age group, by the exact age of 18; wherein females in the delay age group were by the exact age of 21.

Table 3 shows that the percentage of age at marriage for all respondents by different background characteristics, as well as the median age at marriage for various subgroups. It was observed that in the age group below 21 years 92% of males and 91% females in the rural areas got married. On the contrary, in urban areas, 81% of males and 85% of females got married in the same age group. Due to no education or only primary education, or poor family status, and less or no exposure to mass media people were married in the early age group, often below 21 years in places of residence in both the settings. Furthermore in the age group of more than 21 years females were more delayed married as compared to males in both places of residence. The reason behind the delayed age of marriage includes the rich background family with higher education and more exposed to mass media. The Christian and Schedule Tribe (ST) background family had married more at delayed age as compared to the other religions and communities. On the contrary, in the Muslim religion and Hindu Schedule Cast, marriages take place in the place of residence in the early age. While considering the median age at married in the place of residence was varied by the different subgroups. For example, in rural areas, both the male and female median age at married was age 17 years, while in urban areas age was 18. The youth people who were not completed primary education, the median age at marriage for these groups in 17 years for both residences. While, those were completed higher education, median age at married is 21 years for rural residents and 22 years for urban residents. The poorest background family in rural areas most were married at the exact age of 16 years while, in urban residence age at 17 years, on contrary richest background family rural–urban differences median age at married as 21 and 22 years respectively.

**Table 3:** Percentage of age at marriage and the median age at marriage by different background characteristics in place of residence in India

Socio-economic background Characteristics	Rural			Urban			Median age at marriage	
	Less than age 21	21–25	26–30	Less than age 21	21–25	26–30	Rural	Urban
Sex								
Male	92.43	6.37	1.2	88.1	10.67	1.23	17	18
Female	91.12	8.0	0.89	85.5	11.7	2.8	17	18
Higher education								
No Education	93.87	4.98	1.15	93.35	6.25	0.4	17	17
Primary	92.29	6.94	0.76	88.42	9.6	1.98	17	17
Secondary	85.03	13.48	1.49	79.96	16.91	3.13	18	18
Higher Secondary	62.5	31.25	6.25	44.98	46.94	8.08	21	22
Caste								
SC	93.26	5.67	1.08	93.04	6.19	0.77	16	17
ST	90.48	7.95	1.57	79.12	18.47	2.41	18	19
OBC	93.42	5.72	0.86	88.31	10.47	1.21	16	17
Other	91.68	7.08	1.24	84.34	12.45	3.21	17	18
Religions								
Hindu	93.39	5.68	0.92	88.6	9.79	1.61	16	17
Muslim	94.35	4.81	0.84	90.91	8.57	0.52	17	18
Christian	78.78	16.86	4.36	70.51	28.21	1.28	18	20
Other	75.59	21.26	3.15	61.9	33.33	4.76	21.5	22
Wealth quintile								
Poorest	94.33	4.82	0.85	91.56	6.61	1.83	16	17
Poorer	92.55	6.25	1.21	88.36	10.78	0.86	17	17
Middle	90.22	8.26	1.52	89.15	9.01	1.84	18	18
Richer	89.61	9.38	1.01	86.62	11.43	1.95	18	18
Richest	90.22	8.15	1.63	84.46	13.29	2.25	21	22
Exposure to mass media								
Yes	93.79	5.14	1.07	89.23	9.02	1.75	17	18
No	91.22	7.57	1.21	87.15	10.88	1.55	17	17

Table 4 contains the life table estimate prevalence of pre-marital sex among the youth people by the different background characteristics in the place of residence in India. The result suggested that the prevalence of pre-marital sex among the females in early-age married was higher than the male (58.4 vs. 52.8%) in rural residences, while in urban residences differences were narrowed (57.1 vs. 57.0%). The prevalence of pre-marital sex was higher due to delayed age married for both groups in rural and urban residences. The prevalence of unmarried people till 30 years was lower than delayed age married people, but males are more engaged in premarital sex than females for both residences (52.5 vs. 47.7% and 38.8 vs. 22.2%). However, males were slightly more likely to have had premarital sex than those have till unmarried at the age of 30 years; while females were more likely to have premarital sex at delayed age. The percentage of pre-marital sex among the scheduled tribe community was higher than the other social group at an early age. Furthermore, the early age of premarital sex was found in rural areas for those who belonged to the poorest economic family background with low education and Muslim religious community in the country. On the other hand, delayed ages at premarital sex were found in urban residents who had covered higher education, higher exposure to mass media, and the richest economic family respectively.

**Table 4:** Prevalence of premarital sex with 95% confidence interval by different background characteristics in place of residence in India

Socio-economic background Characteristics	RURAL						URBAN					
	Early Married (< 21 year)		Delayed Married (27–30 year)		Unmarried till 30 year		Early Married (< 21 year)		Delayed Married (27–30 year)		Unmarried till 30 year	
	%	95% CI	%	95% CI	%	95% CI	%	95% CI	%	95% CI	%	95% CI
Sex												
Male	52.85	51.69–54.03	62.35	61.22–63.48	52.59	50.99–54.21	57.06	51.98–62.26	72.32	67.58–76.87	38.85	36.49–41.3
Female	58.48	55.69–61.3	68.44	65.78–71.07	47.76	43.92–51.75	57.14	45.4–69.46	77.78	66.92–87.06	22.21	18.26–26.62
Higher Education												
No Education	53.56	52.26–54.86	61.15	59.87–62.42	48.74	43.96–53.76	61.20	58.33–64.09	70.77	68.05–73.43	50.77	39.36–63.36
Primary	55.94	53.32–58.6	67.01	64.50–69.50	56.02	51.12–61.05	61.66	56.83–66.51	73.32	68.83–77.63	49.38	39.13–60.7
Secondary	51.98	49.14–54.89	68.62	65.94–71.27	61.21	59.48–63.08	48.51	44.61–52.57	74.67	71.14–78.07	52.22	49.25–55.27
Higher	41.86	28.81–57.92	72.09	58.35–84.43	15.53	13.10–18.36	31.6	21.21–45.34	71.93	59.99–82.83	9.54	7.65–11.86
Caste												
SC	62.90	60.48–65.32	72.96	70.71–75.16	57.69	54.21–61.22	62.29	57.67–66.93	73.27	68.96–77.41	44.49	38.72–50.7
ST	42.33	40.52–44.19	49.21	47.37–51.08	41.61	39.35–43.94	47.40	42.15–52.95	61.77	56.54–67.04	27.02	23.6–30.83
OBC	59.72	57.88–61.57	70.56	68.84–72.27	62.41	59.72–65.10	59.75	56.66–62.86	76.75	74.03–79.37	43.89	40.17–47.81
Other	56.51	52.71–60.39	69.39	65.78–72.93	51.63	47.0–56.43	52.40	46.60–58.45	69.74	64.22–75.11	28.26	24.15–32.9
Religions												
Hindu	58.29	57.08–59.51	68.35	67.20–69.50	59.10	57.30–60.90	58.94	56.50–61.4	74.5	72.31–76.64	39.44	36.67–42.34
Muslim	59.12	52.04–60.27	66.55	62.61–70.44	64.44	58.88–69.97	59.17	54.45–63.95	72.86	68.48–77.08	44.85	39.17–50.96
Christian	34.41	30.39–36.64	40.5	37.33–43.84	30.69	27.79–33.83	45.16	36.23–55.24	65.38	56.27–74.35	22.07	18.46–26.27
Other	25.24	19.91–31.69	30.48	24.73–37.19	33.57	26.48–41.94	25.0	13.87–42.54	38.89	25.18–56.65	25.0	16.55–36.70
Wealth quintile												
Poorest	51.27	49.44–53.13	57.77	55.96–59.60	64.53	61.83–67.23	53.96	45.93–62.40	65.47	57.57–73.25	67.19	55.67–78.27
Poorer	54.5	52.56–56.45	63.6	61.73–65.48	56.65	53.90–59.44	57.14	51.21–63.22	70.66	65.04–76.08	50.36	42.34–58.98
Middle	55.82	53.57–58.10	68.61	66.48–70.72	43.95	40.91–47.11	63.05	58.93–67.17	75.43	71.68–79.02	41.28	36.29–46.68
Richer	55.39	51.95–58.88	69.07	65.83–72.27	40.60	36.79–44.64	55.35	51.82–58.94	71.12	67.84–74.33	36.17	32.36–40.28
Richest	50.7	44.23–57.54	63.26	56.84–69.67	31.31	26.49–36.77	53.38	48.96–57.92	73.84	69.82–77.71	28.15	25.25–31.31
Exposure to mass media												
Yes	56.24	54.83–57.65	67.37	66.03–68.70	49.21	47.57–50.87	56.76	54.51–59.03	73.09	71.05–75.09	34.9	32.8–37.09
No	50.1	48.44–51.79	57.5	55.85–59.15	63.75	60.42–67.07	57.95	52.45–63.55	67.88	62.58–73.07	57.38	45.45–69.88

Table 5 presents the Adjusted Relative Ricks Ratios (RRR) of premarital sex by different background characteristics in the place of residence in India. The result suggested that early marriage is a reference category, while the relative risk for pre-marital sex in delayed married 1.57 times higher in urban residence as compared to the rural residence. The tendency of premarital sex among the unmarried people was lower and also the negligible difference in residence while in rural areas relative risk for pre-marital sex 0.23 times and urban areas 0.26 times respectively. The females are at a greater risk of premarital sex in urban residences as compared to rural residences in the country with statistical significance (RRR 1.33 vs. 1.30). Earlier a cross-sectional study by Liu et al., (2006) was conducted on premarital sex among vocational students in northern Thailand at aged 15–21 years and suggested female sexual initiation was associated with earlier initiation at a younger age [39]. Because of living away from family, lacking a family members are getting more confidants for premarital sex. Considering education groups with no education as the reference category and the risks of pre-marital sex were higher who had completed secondary and higher secondary than those who completed only primary education. In addition, the higher educated people in urban residences 1.78 times had a higher risk of having premarital sexual intercourse than the rural residence with statically significant (RRR 1.78 vs. 1.66). Thus, “delayed marriage for literate women indicates that they have a long time before getting married. However, a single literate woman is less likely to experience sexual intercourse than an illiterate single woman” [40]. Another interesting finding Muslim religious people in rural residences 1.78 times higher risks of pre-marital sex than urban residents without statically significant in the country. In the context of pre-marital sex with the economic background of the family, the richest people have a higher relative risk of premarital sex compared to the poorest background family for both residences with statically significant in the country. Those people highly exposed to mass media to higher chances to engaged premarital sex as compared to those who have no exposure to any other mass media (radio, television, newspaper) respectively.

**Table 5:** Adjusted Relative Risk Ratios (RRR) of premarital sex by selected characteristics in place of residence in India

Socio-economic background characteristics	RURAL			URBAN		
	RRR	SE	95% CI	RRR	SE	95% CI
Age at Marriage						
Early Marriage®						
Delayed Marriage	0.72**	0.17	0.448–1.146	1.57***	0.70	0.654–3.775
Unmarried	0.23**	0.20	0.04–1.262	0.19*	0.26	0.013–2.780
Sex						
Male**®**						
Female	1.30***	0.25	0.884–1.908	1.33**	0.49	0.644–2.752
Higher Education						
No Education**®**						
Primary	0.82**	0.13	0.604–1.126	0.89	0.32	0.442–1.791
Secondary	0.96**	0.15	0.701–1.303	1.37**	0.43	0.747–2.522
Higher Secondary	1.66***	0.17	0.417–6.589	1.78***	0.41	0.377–8.416
Caste						
SC**®**						
ST	0.46*	0.08	0.321–0.647	2.42	1.25	0.883–6.653
OBC	0.61	0.10	0.438–0.851	1.07	0.36	0.548–2.072
Other	0.65	0.19	0.362–1.171	0.70***	0.32	0.288–1.701
Religions						
Hindu**®**						
Muslim	1.78	0.56	0.965–3.288	1.17	0.37	0.622–2.183
Christian	0.97**	0.24	0.597–1.583	0.50***	0.39	0.105–2.355
Other	0.28**	0.11	0.126–0.625	0.14*	0.20	0.007–2.525
Wealth Quintile						
Poorest**®**						
Poorer	1.00**	0.15	0.742–1.354	1.01*	0.00	1.009–1.011
Middle	1.18**	0.22	0.819–1.686	1.37	0.60	0.583–3.235
Richer	1.75***	0.55	0.941–3.256	1.75**	0.80	0.717–4.291
Richest	1.62***	0.33	0.22–1.771	1.85***	0.75	0.453–4.003
Exposure to mass media						
No**®**						
Yes	1.28*	0.18	0.969–1.70	2.07***	0.74	1.029–4.159

***p < 0.01; **p < 0.05; *p < 0.1; ® *Reference category*

## Discussion

The result of the analysis indicates that premarital sex is highly determined by the age of marriage of the youth people in the residences. Almost all individuals of both sexes have intercourse before marrying. Available literature suggested that “the prevalence of premarital sex is positively associated with the delayed age of marriage for men and women. Due to poverty, financial implications, and emotional attachment, most females are bound to marry late. This has increased the divorce rate due to various reasons that are post effects of late marriage” [41]. The result of the study highlighted that in rural areas mean age of female marriage is 21.8 years whereas in urban residences 23.4 years. Previous research on sub-Saharan African countries suggested that “the greater decline in child marriage compared to sexual debut before age 18 implies an increase in premarital sex” [42]. On contrary, “the slower decline in childbearing compared to marriage and sexual debut among adolescent girls indicate the inadequate access to modern contraceptives and other ASRH services for sexually active single adolescents” [43, 44]. Further, it is also found that “girls with primary or less education are much more likely to be married early as well as have their first child before the age of 15 than those with secondary or higher education. Early marriage and childbearing were much more common in rural areas with the poorest family than in urban areas with the richest family. The highly educated people may have better knowledge of access to contraception, more awareness in earlier pregnancies while in rural younger people lack in education. Due to lower knowledge of reproductive health and sexuality they commence their adult life course earlier and thus engage in long-term relationships that are expected to lead to marriage earlier in life. These gaps persisted or even increased over time in the residences. These adverse trends highlight that the disadvantages associated with these outcomes are increasingly concentrated within already vulnerable poor and rural girls” [45]. Similarly, urban–rural ratio and rich–poor disparities in girls with less education led to increasing and persistent long-term inequalities in age-sex reproductive health in different regions. It is also confirmed that “adolescent girls living in rural areas and the poorest households report higher prevalence rates as compared to rich urban households” [46]. Differences in premarital sex between urban and rural residences by young people were contradicted by many scholars. For instance, a few studies reported “premarital sex was more common among rural young people” [47, 48] and, others indicating that have identified prevalence of sexual activity more among those living in urban areas [49]. But these researches have highlighted that males initiated sexual intercourse at an earlier age than females in rural residences, whereas in urban residences delay aged of premarital sex is common among female groups. On contrary, unmarried people till 30 years have lower prevalence of pre-marital sex than those were married latter. This might be explaining in age heaping in older cohort. The earlier study also suggested that “estimates of underreporting are substantially higher for females, perhaps indicative of the different standards and taboos that are applied to young men and women in their societies” [50–53]. Sexual initiation was also associated with having a non-agriculture background, peer groups, and using alcohol consumption [39]. The growing number of the literature suggested that the current use of alcohol, tobacco, and including “peer pressure as the main factors that motivated them to initiate sexual activity among males, while having a poor relationship with parents, exposed to family conflict, and currently living with parents and being an income earner was the strongest association with premarital sex for females” [54–56]. The family was an important influence on premarital sex among the female, some studies reported that “lack of parental control, as well as very strict parenting, were also associated with sex before marriage [57]. Because unintended pregnancy were more likely to be reported among unmarried females in society as compared to married women [58]. This unintended pregnancy was associated with a higher risk of sexual behaviour with multiple partners and forced sex, a lower level of sexual reproductive health knowledge, without the use of contraception, and a lack of parental supervision [37, 59, 60].

Furthermore, unequal access to education and health services are also the reasons for increasing trend of premarital sex in different places of residence. Girls becoming pregnant or marry early are responsible for the drop outs of school and therefore naturally their future opportunities were limited. Further, it is also found that “higher educated unmarried adults in India were more likely to have multiple partners in premarital sexual relations” [15]. Further, “education has created more time and opportunities for young people to adopt liberalized sexual norms and get involved in intimate (sexual) relationships before marriage” [61, 62]. The prior research also suggests that “educated mothers have greater educational aspirations for their children and may discourage them from engaging in activities, including pre-marital sexual activity that might affect the realization of those aspirations” [29].

The richest and richer wealth quintiles are more likely to have multiple premarital sex partners at a delayed age as compared to the poorer and poorest wealth quintiles at an early age. As dating generally requires gifts and parties, which youths from advantaged households can afford. Thereby there is an increase in the risks of premarital intercourse especially among girls from wealthier families. On the other hand, in the poverty driven households, socio-economic status is an important criterion because male youth expect gifts from females of advantaged households. Conversely, female youth from poor households are in need and thus they don’t share. In a nutshell, at the same levels of beauty/attractiveness, both female and male youth from rich families are favored [63].

“The major role of mass media in the formation of multiple partners in premarital sexual relationships was described in the place of residence” [64]. Reporting premarital sex at an early age is higher among rural residents those who have no regular exposure to media, while in contrast percentage of premarital sex among the late married people was higher despite having constant exposure to media in urban residences. Previous study also mentioned that those people never watch western television, listen to pop music or once a week listen/ watch are less likely to engaged in pre-marital sexually activities, while people who watch/listen at several times a week are more likely to have done pre-marital sex [65] and these prevalence are more common urban residence as compared to rural residence respectively. The sex is not bad for the health. The Robert Malthus also mentioned in his preventive check the passion between the sexes appears to be fairly constant but “when a general corruption of morals with regard to sex pervades all classes of society, its effects must necessarily be to poison the springs of domestic happiness, to weaken conjugal parental affection…” thus sex in early ages and sex with multiple partner also violent for HIV/AIDS. Knowledge of HIV/AIDS is highly relevant for premarital sex among both males and females in place of residence. Rural residents lack in knowledge of HIV/AIDS, thus, they sexual debut at an early age, on the contrary, in urban residents, detailed knowledge of HIV/ AIDS is increased, and therefore, reported delayed premarital sex.

## Conclusion

Findings of this study reflect on the rural–urban differences in age at marriage and premarital sex in the country. It is asserted that marriages get delayed among the educated and richest households. The pattern of delayed marriages and premarital sex at the state level might differ in large scale [66]. Therefore, it is difficult to draw any firm conclusion pertaining to the states based on the present research, because of rural–urban disparity and poorest to richest wealth quintile from one state to another winding the gap age at marriage and delayed premarital sex. As mentioned earlier that the educated and richest unmarried people having premarital sex are more likely to have multiple sexual partners in urban residences as compared to the rural residence [41]. Policy-makers and programs needs to take action to for sexual health status and need to provide “services to unmarried young women, supplying condoms, decriminalizing commercial sex and homosexual activity, and prosecuting people who commit sexual violence are likely to be beneficial rather than detrimental” [67, 68]. These programs also need to take the emphasize of rural girls’ empowerment for making proper decisions and preparing them for marriage at the appropriate manner and time. Apart from that improve the inclusion of disadvantage unmarried youth people for sexually reproductive health research, particularly those are out of school, economically disadvantage and marginalized group.

## Data Availability

This research work was performed based on secondary data which is freely available upon request at IIPS, India website (Source of data: http://rchiips.org/NFHS/NFHS4Reports/India.pdf).
